# Clinical benefit of treatment with eribulin mesylate for metastatic triple‐negative breast cancer: Long‐term outcomes of patients treated in the US community oncology setting

**DOI:** 10.1002/cam4.1705

**Published:** 2018-07-31

**Authors:** Sarah S. Mougalian, Ronda Copher, Jonathan K. Kish, Lindsay McAllister, Zhixiao Wang, Mary Broscious, David Garofalo, Janna Radtchenko, Bruce A. Feinberg

**Affiliations:** ^1^ Yale Cancer Center Yale School of Medicine New Haven Connecticut; ^2^ Eisai Inc. Woodcliff Lake New Jersey; ^3^ Cardinal Health Specialty Solutions Dallas Texas

**Keywords:** eribulin, metastatic breast cancer, real‐world, survival, triple negative

## Abstract

**Introduction:**

Real‐world data are critical to demonstrate the consistency of evidence and external generalizability of randomized controlled trials (RCTs). This study examined characteristics and outcomes of metastatic triple‐negative breast cancer (mTNBC) patients treated with eribulin mesylate at community oncology practices in the United States.

**Methods:**

Physicians identified mTNBC patients initiating eribulin between 1 January 2011 and 1 January 2014 and abstracted data into an electronic case report form (eCRF). Eribulin treatment in the metastatic setting was categorized as early use (EU, first‐/second‐line) and late use (LU, third‐line or later). Patient characteristics, overall survival (OS), disease response (per treating physician), and adverse events (AEs) rates in each group, respectively, are reported.

**Results:**

Overall 252 eCRFs were completed: 125 (49.6%) EU and 127 (50.4%) LU. The median age at metastatic diagnosis was 53 years and 42.1% were stage IV at their initial diagnosis. The median duration of follow‐up from the initiation of first‐line treatment was 24 months. Rates of disease response (complete or partial per treating physician) were 69.9% in the EU group and 48.8% in the LU group. The five most commonly reported adverse events during eribulin were as follows: fatigue (65.1%), weakness (40.1%), decreased appetite (32.5%), neutropenia (31.0%), and leukopenia (27.4%). Discontinuation of eribulin due to AE was observed in 4.0% of patients. Median OS from initiation of eribulin was 23.0 months (95% CI: 18.7‐27.3) among EU and 14.7 (95% CI: 12.6‐16.9) among LU.

**Conclusion:**

In the real‐world eribulin‐treated mTNBC, patients have more sites of metastatic disease and exposure to greater numbers of prior therapies compared to RCTs. The median OS of 14.7 months among LU patients is consistent with, and slightly longer than the 13.1 months and 14.4 months reported in the EMBRACE and Study 301 clinical trials, respectively.

## 1 INTRODUCTION

Breast cancer patients who are both hormone receptor (HR)‐negative and HER2‐negative account for 15%‐20% of all breast cancers diagnosed in the United States (US).^1^, ^2^ Compared to patients with HR‐positive or HER2‐positive tumors where average survival exceeds 50 months survival for women with metastatic triple‐negative breast cancer (mTNBC) is considerably shorter ranging from 11 to 17.8 months.^3^, ^4^, ^5^, ^6^ The lower survival for mTNBC patients reflects the limited treatment options available when endocrine or HER‐2 targeting therapy is not an option. As a result, sequential chemotherapy remains the mainstay of treatment for patients with mTNBC and has not dramatically altered the prognosis for these patients.^7^, ^8^, ^9^

Eribulin mesylate (eribulin) is a microtubule inhibitor approved in the US as treatment for women with metastatic breast cancer (mBC) who have received at least two prior chemotherapy regimens including an anthracycline and a taxane in either the metastatic or adjuvant setting. This approval was based on the findings from the EMBRACE trial in which eribulin significantly improved survival by 2.5 months over treatment with physician's choice (TPC) in women with 2 to 5 prior lines of therapy (eribulin arm, median overall survival (OS) = 13.1 months, TPC arm = 10.6 months; *P* = 0.041).^10^ A second phase III study (Study 301) comparing eribulin to capecitabine in women with mBC previously treated with an anthracycline and taxane who had received 0‐2 prior lines failed to meet its primary endpoint finding that no statistically significant reduction in the risk of death among all patients (hazard ratio [HR] = 0.88; 95% CI: 0.77‐1.00).^11^ However, a prespecified subgroup analysis of mTNBC patients showed a statistically significant improvement in survival of 5 months in the eribulin‐treated group (eribulin arm median OS = 14.4 months, capecitabine arm = 9.4 months; HR = 0.70; 95% CI: 0.55‐0.91).^12^ Subsequently, a pooled analysis of all mTNBC patients enrolled in either the EMBRACE and Study 301 found a 26% reduction in the risk of death in the eribulin‐treated patients (HR = 0.74; 95% CI: 0.60‐0.92, *P* = 0.006).^13^

While eribulin is an established therapeutic option for patients with mBC, the real‐world clinical benefit and safety of eribulin therapy for patients with mTNBC when used at different points of the treatment journey have not been reported. This research sought to describe real‐world patient characteristics, disease response, toxicity, and OS for mTNBC patients receiving care at community oncology practices in the US and treated with eribulin as their first‐ or second‐line of therapy (“early use”) or when used as a third‐line or greater (“late use”) treatment, respectively.

## 2 METHODS

Patients were identified by providers in the Cardinal Health Oncology Provider Extended Network (OPEN), a community of over 7000 oncologists, hematologists, and urologists from across the US. OPEN is comprised primarily of community practitioners in both single‐physician and large group practices and membership is not restricted to select sites or to providers at practices who are members of any specific group purchasing organization. Providers abstracted data for patients they personally managed or treated who met the following criteria: female and biopsy confirmed diagnosis of mBC, initiated treatment with eribulin between 1 January 2011 and 1 January 2014, and triple negative defined as ER <1%, PR <1%, and HER2‐negative according to current American Society of Clinical Oncology and College of American Pathologists guidelines.^14^ Patients participating in any interventional clinical trials prior to the start of eribulin therapy, those patients receiving treatment for a second primary malignancy while under the care of the responding physician, and those less than 18 years of age at the initiation of eribulin were not eligible. Providers were instructed to randomly select up to 10 patients meeting these criteria and complete an electronic case report forms (eCRFs) for each eligible patient. The number and characteristics of eligible patients treated by the physician who were not selected were not captured during data collection. The eCRF captured the following data: patient demographics, clinical characteristics at diagnosis and eribulin initiation (stage at diagnosis, site of metastases, Eastern Cooperative Oncology Group performance status [ECOG‐PS]), drug regimens received up to initiation (including neoadjuvant/adjuvant) of eribulin by line of therapy (LOT) and total LOTs (dates of initiation/discontinuation of each LOT, disease response (per treating physician), adverse events (AEs) during eribulin treatment (only those AEs reported in the eribulin trials), and date of death (or last follow‐up). Disease response was per the treating provider's interpretation and not assessed by independent review based on RECIST criteria.

All submitted eCRFs were reviewed by Cardinal Health clinical research staff for quality control. Items such as implausible dates (eg, date of death before last date of treatment), lab or radiology results considered inconsistent with known clinical parameters, or nonstandard treatments were flagged and providers were contacted for data validation. In addition, a random sample of 10% of all submitted eCRFs was validated through provider follow‐up. Data which could not be validated were not included in the final study analysis.

Two cohorts were created according to the LOT at first initiation of eribulin: the early use (EU) cohort was defined as receipt of eribulin as first‐ or second‐line therapy for mTNBC. The late use (LU) cohort was defined as the receipt of eribulin in third‐line or greater. The target sample size for the study was 250 patients evenly distributed between the EU and LU cohorts and was selected based on the resources available for chart data abstraction (physicians participating in the research were provided an honoraria payment based on the total number of hours spent on data abstraction). Patient demographic and clinical characteristics were compared between the two groups to examine predictors of early or late use. Toxicity rates, disease response, and OS were not compared between the groups as the study was neither designed nor powered to evaluate these differences. Results are presented through descriptive analyses including frequencies, proportions, means and standard deviations for categorical and continuous variables and outcomes, respectively. Complete response (CR) and partial response (PR) were classified as tumor response. Median OS was assessed using the Kaplan‐Meier method from the initiation of eribulin until date of death in the EU and LU cohorts, respectively, to approximate survival from randomization reported in the eribulin RCTs (as opposed to from the date of metastatic diagnosis). Patients were censored on the date of last follow‐up if a death date was not provided. Patients with missing eribulin start dates were not considered in the analysis of OS. All analyses were conducted using Statistical Analysis Software v9.4 (SAS Institute, Cary, NC).

A central institutional review board (Western IRB, Puyallup, Washington) reviewed the study protocol and case report formed and deemed the study exempt from full review. A waiver of informed consent was obtained for the study. Data collection began on 18 March 2016 and concluded on 1 September 2016.

## 3 RESULTS

### 3.1 Demographics and clinical characteristics

Fifty‐eight providers completed data extraction for a total of 252 mTNBC patients including 125 (49.6%) EU and 127 (50.4%) LU. Demographic characteristics are shown in Table 1. The median age at diagnosis for all patients was 53 years; while the mean age at initiation of eribulin was older for EU at 57.1 vs LU at 54.4 years (*P* = 0.046). Overall, 42.1% of patients had stage IV disease at diagnosis and LU were more likely to have an initial diagnosis of distant metastatic disease (51.2% vs 32.8%, *P* = 0.02). The most frequent sites of metastasis among all patients were: bone (59.9%), lung (50.4%), lymph nodes (44.8%), and liver (43.7%) with LU patients having more frequent bone metastases (76.4% vs 60.8%, *P* = 0.008). Nearly all patients (95.6%) had an ECOG‐PS score of 0/1 at the initiation of eribulin therapy with no significant difference observed between EU and LU (*P* = 0.641).

**Table 1:** Demographics and clinical characteristics of patients

	All eribulin patients Any of line of therapy (n = 252)		Early eribulin users Line of therapy 1 or 2 (n = 125)		Late eribulin users Line of therapy ≥3 (n = 127)		*P*‐value
Mean age at diagnosis of mBC (mean, SD)	53.3	10.9	54.1	10.1	52.2	11.9	0.173
Median	53		54.0		52		
Mean age at eribulin initiation (SD)	55.7	10.8	57.1	10.6	54.4	10.8	0.046
Median	55.5		57		54		
Region (n, %)							
South	89	35.3	36	28.8	53	41.7	0.140
West	67	26.6	36	28.8	31	24.4	
Midwest	63	25.0	37	29.6	26	20.5	
Northeast	33	13.1	16	12.8	17	13.4	
Stage at diagnosis (n, %)							
Stage I	9	3.6	2	1.6	7	5.5	0.020
Stage IIA	17	6.7	10	8.0	7	5.5	
Stage IIB	43	17.1	23	18.4	20	15.7	
Stage IIIA	42	16.7	29	23.2	13	10.2	
Stage IIIB	17	6.7	10	8.0	7	5.5	
Stage IIIC	14	5.6	7	5.6	7	5.5	
Stage IV	106	42.1	41	32.8	65	51.2	
Unknown	4	1.6	3	2.4	1	0.8	
Sites of metastatic disease at initiation of eribulin (n, %)							
Bone	151	59.9	70	56.0	81	63.8	0.008
Liver	110	43.7	60	48.0	50	39.4	0.312
Lymph nodes	113	44.8	62	49.6	51	40.2	0.524
Lung	127	50.4	67	53.6	60	47.2	0.177
Brain	11	4.4	6	4.8	5	3.9	0.737
Other	14	5.6	5	4.0	9	7.1	0.443
ECOG‐PS at initiation of eribulin (n, %)							
0	40	15.9	27	21.6	13	10.2	0.641
1	156	61.9	81	64.8	75	59.1	
2	56	22.2	17	13.6	39	30.7	
3/4	0	0.0	0	0.0	0	0.0	

Table 2 shows total LOTs, length of follow‐up, LOT at initiation of eribulin, and patient status at the end of the study period. The median length of follow‐up from the initiation of first‐line therapy was 24 months. Chemotherapy in the neo/adjuvant setting was received by 54.0% of all patients including 63.2% of EU and 44.9% of LU. Among patients who received neo/adjuvant treatment, 86.0% had received treatment with both an anthracycline and a taxane. Among 125 EU, 20.8% initiated eribulin as first‐line metastatic treatment and the remainder in second‐line. For LU, 73.2% initiated eribulin in the third‐line, 19.7% in fourth line, and 7.1% in fifth line or greater. At the end of the study period, 192 (84.6%) of all patients were deceased including 72.0% of EU (n = 90) and 80.3% of LU (n = 102).

**Table 2:** Clinical characteristics at diagnosis, initiation of eribulin treatment, and end of study period

	All eribulin patients Any of line of therapy (n = 252)		Early eribulin users Line of therapy 1 or 2 (n = 125)		Late eribulin users Line of therapy ≥3 (n = 127)	
Months of follow‐up from LOT 1 initiation (mean, SD)^a^	26.6	12.8	24.0	12.2	29.1	12.8
Total lines of therapy (n, %)						
1	9	7.2	9	7.2	0	0.0
2	39	15.5	39	31.2	0	0.0
3	91	36.1	43	34.4	48	37.8
4	65	25.8	24	19.2	41	32.3
5	38	15.1	9	7.2	29	22.8
6	10	4.0	1	0. 8	9	7.1
Line of therapy at initiation of eribulin (n, %)						
1	26	10.3	26	20.8	0	0.0
2	99	39.3	99	79.2	0	0.0
3	93	36.9	0	0.0	93	73.2
4	25	9.9	0	0.0	25	19.7
5	8	3.2	0	0.0	8	6.3
6	1	0.4	0	0.0	1	0.8
Any neo/adjuvant treatment with either anthracycline or taxane (n, %)	136	54.0	79	63.2	57	44.9
Neo/adjuvant treatment with anthracycline^b^	131	96.3	77	97.5	54	94.7
Neo/adjuvant treatment with taxane^b^	122	89.7	72	91.1	50	87.8
Neo/adjuvant treatment with both anthracycline and taxane^b^	117	86.0	70	88.6	47	82.5
Status at end of study period (n, %)						
Deceased	192	76.2	90	72.0	102	80.3
Currently on treatment	18	7.1	11	8.8	7	5.5
Receiving palliative care	18	7.1	5	4.0	13	10.2
Unknown/lost to follow‐up	22	8.7	17	13.6	5	3.9
Other (surveillance, clinical trial)	2	0.8	2	1.6	0	0.0

203 patients with known first date of treatment and last follow‐up date were included.

Proportion was out of number of patients who were treated with either an anthracycline or a taxane; n = 136 all patients, n = 79 EU, and n = 57 LU.

### 3.2 Outcomes of eribulin therapy

Table 3 reports the duration of eribulin treatment, rationale for treatment discontinuation, provider assessment of disease response, and rate of AEs. Among the 225 (89.3%) patients with a validated eribulin treatment initiation and discontinuation dates, the mean duration of eribulin treatment was 6.0 months (SD = 3.8) among EU and 5.3 months (SD = 4.7) among LU disease response (CR or PR) was 69.9% for EU and 48.8% for LU. Among patients with a duration of eribulin therapy of <6 months (n = 169), response rates were 58.6% for EU and 42.6% for LU. Among patients with duration of eribulin therapy ≥6 months, response rates were 84.0% and 66.7% in the EU and LU groups, respectively. Overall, providers indicated that 90.5% of patients treated with eribulin discontinued therapy due to disease progression, 8.0% due to patient request or financial challenges, and 4.0% due to an AE (rationale not mutually exclusive). During the course of treatment, the most common AEs reported among EU patients were as follows: fatigue (64.8%), neutropenia (36.8%), weakness (36.8%), leukopenia (32.0%), and peripheral neuropathy (25.6%). Among LU, the most frequently diagnosed AEs were: fatigue (65.4%), weakness (43.3%), decreased appetite (40.2%), peripheral neuropathy (37.0%), and alopecia (36.2%).

**Table 3:** Tumor response (per provider determination^b^) and occurrence of adverse events during eribulin therapy

	All eribulin patients Any of line of therapy (n = 252)		Early eribulin users Line of therapy 1 or 2 (n = 125)		Late eribulin users Line of therapy ≥3 (n = 127)	
Duration of treatment in months (mean, SD)^a^	5.6	4.3	6.0	3.8	5.3	4.7
Disease response^b^						
Response (complete/partial)	148	58.7	86	69.9	62	48.8
Stable disease	73	29.0	28	22.8	45	35.4
Progressive disease	29	11.5	9	7.3	20	15.7
Disease response (<6 mo eribulin treatment duration, n = 75 EU and n = 94 LU)^b^						
Response (complete/partial)	84	33.3	44	58.6	40	42.6
Stable disease	55	21.8	20	26.7	35	37.2
Progressive disease	28	11.1	9	12.0	19	20.2
Unknown	2	0.8	2	2.7	0/94	0.0
Disease response (≥6 mo eribulin treatment duration, n = 50 EU and n = 33 LU)^b^						
Response (complete/partial)	64/83	77.1	42/50	84.0	22/33	66.7
Stable disease	18/83	21.7	8/50	16.0	10	30.3
Progressive disease	1/83	1.2	0/50	0.0	1	3.0
Unknown	0/83	0.0	0/50	0.0	0	0.0
Rationale for eribulin discontinuation						
Tumor progression	228	90.5	106	84.8	122	96.1
Adverse event	10	4.0	8	6.4	2	1.6
Patient request/financial challenges	20	8.0	18	14.4	2	1.6
Other	9	3.6	5	4.0	4	3.1
Adverse events diagnosed during eribulin treatment						
Neutropenia (ANC <1000 cells/mL)	78	31.0	46	36.8	32	25.2
Leucopenia (WBC <4000 cells/mL)	69	27.4	40	32.0	29	22.8
Alopecia	77	30.6	31	24.8	46	36.2
Lymphopenia (<1000 cells/mL)	15	6.0	6	4.8	9	7.1
Fatigue	164	65.1	81	64.8	83	65.4
Decreased appetite	82	32.5	31	24.8	51	40.2
Nausea and vomiting	59	23.4	26	20.8	33	26.0
Stomatitis	30	11.9	13	10.4	17	13.4
Taste abnormality	42	16.7	22	17.6	20	15.7
Decreased hemoglobin (<10 mg/dL)	52	20.6	21	16.8	31	24.4
Increased AST/ALT (GOT/GPT)	21	8.3	10	8.0	11	8.7
Increased CK (CPK)	1	0.4	0	0.0	1	0.8
Weakness	101	40.1	46	36.8	55	43.3
Fever	7	2.8	3	2.4	4	3.1
Peripheral neuropathy	79	31.3	32	25.6	47	37.0

Provider may base their interpretation of the patients response on a variety of factors, including, but not limited to RECIST criteria. As such, response should not be considered per RECIST.

a Includes 109 EU and 116 LU with known eribulin initiation and discontinuation dates.

Two patients with unknown response were not included in EU response proportion (total n = 123).

75 EU and 94 LU patients had a duration of eribulin treatment <6 mo; proportion was reported respectively.

b 50 EU and 33 LU patients had a duration of eribulin treatment ≥6 mo; proportion was reported respectively.

### 3.3 Overall survival from initiation of eribulin

**Figure 1:**
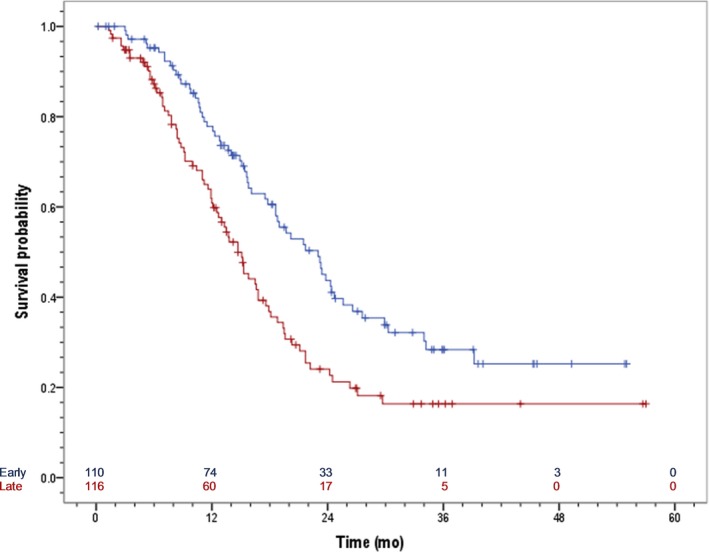
Overall survival from initiation of eribulin stratified by early (LOT 1/2) vs late (LOT 3+) use of eribulin. Median OS EU **:** 23.0 mo (95% CI: 18.7‐27.3). Median OS LU: 14.7 mo (95% CI: 12.6‐16.9)

Survival analysis was conducted using the 226 patients (89.6%) with complete data for eribulin treatment initiation, last date of follow‐up or date of death. Of the 226 patients, 137 deaths (60.6%) were recorded and 89 patients were censored. For the EU cohort, the median OS from the initiation of eribulin was 23.0 months (95% CI: 18.7‐27.3); for the LU cohort, the median OS from the initiation of eribulin was 14.7 months (95% CI: 12.6‐16.9) (Figure 1). Median OS from the initiation of eribulin for all patients was 17.6 months (95% CI: 15.3‐19.9; data not shown).

## 4 DISCUSSION

Real‐world postmarketing authorization studies of novel therapies often reveal heterogeneity in the population of patients receiving a novel agent compared to the randomized clinical trials (RCTs). With the major limitations of RCTs being the highly selected patient population, real‐world data is critical to demonstrate the consistency of evidence and external generalizability of RCTs. This retrospective chart review expands upon the findings of the pivotal eribulin RCTs as the first large community‐based assessment of outcomes among patients with mTNBC in the US. Overall, our findings are consistent with those RCTs and other observational research. We observed that patients treated in the real‐world were more diverse than those in the pivotal eribulin RCTs in terms of ethnic origin, ECOG‐PS, sites of metastatic disease, and treatments received prior to eribulin; however, these difference did not markedly alter the efficacy of eribulin when compared to estimates of survival from the RCTs.

We observed a median OS among all mTNBC patients from initiation of eribulin of 23.0 months among EU (either first line or with one prior line of therapy in the metastatic setting). In comparison, the median OS in the phase III trial of eribulin compared to capecitabine (Study 301) for patients with mTNBC and 0‐2 prior lines of therapy (71.4% or eribulin‐treated patients with 0‐1 prior LOT and 28.5% with ≥2 LOTs) was 14.4 months in the eribulin arm and 9.4 months in the capecitabine arm (*P* = 0.01).^11^ For LU (at least two prior LOT in the metastatic setting) we observed a median OS of 14.7 months, slightly longer from than the 13.1 months observed in the EMBRACE. In comparison the EMBRACE study included HR‐positive patients, and, like Study 301, estimated OS from the date of randomization. To our knowledge, only one other observational, real‐world retrospective cohort study, conducted in Italy, evaluated survival following eribulin therapy. In this study which included 14 mTNBC patients (out of a sample of 133 eribulin‐treated patients), the observed median OS was 14.3 months.^15^

Tumor response (CR or PR), as reported by the providers without independent verification was observed in 69.9% of EU and 48.6% of LU. These rates were higher in comparison to both eribulin RCTs and other retrospective observational cohort studies. In the EMBRACE study and Study 301, the objective response rates were 12.0% and 11.0%, respectively. Other observational studies have shown overall response rates of 21.4% and 29.0%.^15^, ^16^ Our results are more similar to the observed clinical benefit rates (including CR, PR and stable disease for >6 months) reported in the eribulin RCTs which were 56.6% (Study 301) and 67.5% (EMBRACE). However, when including stable disease the rate of clinical benefit observed in our research increases further to 84.0% of EU and 67.7% of LU (among patients with a duration of eribulin therapy ≥6 months). Other observational research has shown a clinical benefit rate of 35.7% (response or stable disease for ≥6 months).^15^ Disease response in our observational study was likely overestimated due to several factors. First, independent review of tumor response was not planned or conducted. Next, providers may have classified a slowed rate of tumor growth as a disease response and not relied on objective measurements. Third, providers may have seen a mixed response and continued with therapy indicating that a patient did respond but the response was temporary. Finally, as an observational study, clinical measurement does not occur at set intervals following the initiation of treatment. As such response reporting may be upwardly biased due to information bias (ie, that scan results were not available at the time of the data abstraction and providers subjectively gauged disease response).

Across both EU and LU cohorts, the most commonly reported AEs during eribulin treatment included fatigue and weakness, neutropenia, peripheral neuropathy, and alopecia. Comparatively, our data are consistent with other observation research in which fatigue, (37.6%), alopecia (27.8%), gastrointestinal toxicity (18.0%), neurotoxicity (18.0%), and arthralgia (12.7%) were the five most commonly observed toxicities. Peripheral neuropathy rates with eribulin were higher in our study compared to the EMBRACE study and may reflect pre‐existing illness rather than incident diagnosis during eribulin treatment. However, neutropenia rates were lower (EU = 36.8% and LU = 25.2%) than reported in RCTs (all grades = 54.2% in 301 Study and 52.0% in EMBRACE). This difference likely reflects limitations of data capture in that only clinically significant events may be recorded in the patient record, as well as the use of supportive care agents such as growth factors to treat low‐grade neutropenia.

### 4.1 Limitations

As a retrospective study using physician‐lead data abstraction from patient records this research has several key limitations reflecting potential biases such as information bias due to availability and quality of data contained within the patient charts (including the providers interpretation of disease response) and selection bias. In regard to the former providers estimates of disease, response may be overestimated given providers were not asked to explain the rationale for assigning a disease response level, use the RECIST criteria to calculate response, or to provide baseline and best response tumor measurements for a retrospective, independent calculation of disease response via RECIST criteria. Similarly, AEs related to eribulin therapy and therapy discontinuation were collected; however, grades of AEs were not collected due to the lack of grading that may occur in the course of routine clinical practice. Moreover, AEs that did not reach a level of clinical significance to require intervention or be noted in the chart may not be well documented by physicians.

In regard to selection bias, a control or comparison arm was not evaluated in this study. All patients had to receive eribulin for inclusion in the research dataset. As such, estimates of OS, disease response or AEs in a non‐eribulin‐treated patient were not available to contextualize our findings. Therefore, the degree to which these estimates are confounded by unknown patient or provider‐level factors cannot be determined. Additionally, the total number of eligible patients who were not submitted or the frequency of charts discarded with data that could not be validated was not captured limiting our ability to detect any systematic bias in the selected patients. Finally, we acknowledge that there are significant clinical and treatment differences between the EU and LU groups as was described. Therefore, the authors note that comparisons of outcomes between the EU and LU groups, or consideration of the eribulin‐treated population overall, in terms of the clinical benefit or safety should not be made.

## 5 CONCLUSIONS

This is the first large study of eribulin use among real‐world mTNBC patients treated in the US. This study found that eribulin is used in a more heterogeneous population than those included in eribulin RCTs in terms of ethnic origin, ECOG‐PS, and sites of metastatic disease. Moreover, we found significant heterogeneity in the characteristics of patients treated with eribulin early in the course of treatment vs late. In comparison with other evidence, we did not observe a reduction in clinical benefits of eribulin therapy in terms of OS or changes in the safety or intolerability profile when used either early or late in the course of treatment. Future comparative studies are needed to generate real‐world evidence to compare outcomes of mTNBC patients treated with eribulin vs other therapies. Additionally, future research should consider including an independent tumor response assessment. Finally, the impact of therapeutic sequencing prior to and posteribulin therapy should be addressed. There is lack of evidence on efficacy based on treatment sequencing and novel therapies, including eribulin and more recently cytokine‐dependent kinase inhibitors, may have a significant impact on clinical benefit when used in an optimal treatment sequence.

## CONFLICT OF INTEREST

Copher is an employee of Eisai Inc. Wang was an employee of Eisai at the time of the research and manuscript drafting. Mougalian received funding from Eisai during the study and manuscript preparation. Kish, McAllister, Brosicous, Radtchenko, and Feinberg are employees of Cardinal Health which received funding from Eisai to conduct this research and prepare manuscript. Garofalo was an employee of Cardinal Health during data collection and analysis.
